# Fe-Based Nano-Materials in Catalysis

**DOI:** 10.3390/ma11050831

**Published:** 2018-05-17

**Authors:** Stavros Alexandros Theofanidis, Vladimir V. Galvita, Christos Konstantopoulos, Hilde Poelman, Guy B. Marin

**Affiliations:** 1 Laboratory for Chemical Technology, Ghent University, Technologiepark 914, B-9052 Ghent, Belgium; StavrosAlexandros.Theofanidis@Ugent.be (S.A.T.); Hilde.Poelman@UGent.be (H.P.); Guy.Marin@UGent.be (G.B.M.); 2 Department of Engineering, University of Campania “Luigi Vanvitelli”, Via Roma 29, 81031 Aversa (CE), Italy; konstantopoulos.christos@gmail.com

**Keywords:** role of iron, CO_2_ utilization, chemical looping, nano-alloys, carbon, hydrocarbon conversion, dehydrogenation

## Abstract

The role of iron in view of its further utilization in chemical processes is presented, based on current knowledge of its properties. The addition of iron to a catalyst provides redox functionality, enhancing its resistance to carbon deposition. FeO_x_ species can be formed in the presence of an oxidizing agent, such as CO_2_, H_2_O or O_2_, during reaction, which can further react via a redox mechanism with the carbon deposits. This can be exploited in the synthesis of active and stable catalysts for several processes, such as syngas and chemicals production, catalytic oxidation in exhaust converters, etc. Iron is considered an important promoter or co-catalyst, due to its high availability and low toxicity that can enhance the overall catalytic performance. However, its operation is more subtle and diverse than first sight reveals. Hence, iron and its oxides start to become a hot topic for more scientists and their findings are most promising. The scope of this article is to provide a review on iron/iron-oxide containing catalytic systems, including experimental and theoretical evidence, highlighting their properties mainly in view of syngas production, chemical looping, methane decomposition for carbon nanotubes production and propane dehydrogenation, over the last decade. The main focus goes to Fe-containing nano-alloys and specifically to the Fe–Ni nano-alloy, which is a very versatile material.

## 1. Introduction and Motivation

**Figure 1:**
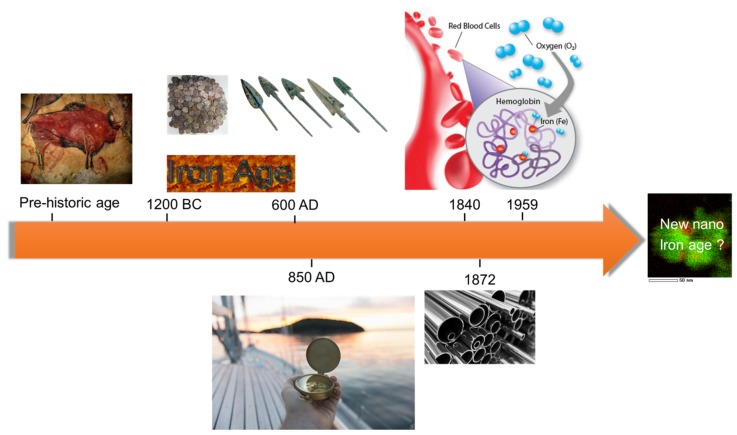
Use of iron/iron oxides throughout mankind [6,7,8].

Iron is one of the most abundant elements in the earth’s crust composing 5% of it, and iron oxides have proven to be valuable materials to mankind over the years, starting from the pre-historic age where iron oxide containing ochre pigments were used to decorate cave walls (Figure 1). Fe_3_O_4_ containing rocks were man’s first experience with magnetism, while compass-like instruments based on Fe_3_O_4_ were already exploited for religious purposes in China around 200 BC [1]. The development of Fe_3_O_4_-based compasses for navigation occurred in Europe approximately around 850 AD. Throughout the 20th century, iron oxides were at the forefront of discovery in science. For example, Fe_3_O_4_ as Fe^2+^Fe^3+^_2_O^2−^_4_ was one of the first spinel structures solved by Bragg in 1915 [2] and Verwey discovered one of the first metal–insulator transitions in Fe_3_O_4_ in 1939. 

Iron is involved in several biological processes. Proteins containing iron can be found in all living organisms [3,4]. In humans, an iron–protein, hemoglobin, is responsible for oxygen transport from the lungs to the rest of the body and for the blood color (Figure 1). Iron oxides, like Fe_3_O_4_, aid the navigation of magnetotactic bacteria [5], and it is thought that they play a similar role in the beaks of homing pigeons, while they have also been discovered in the human brain and other body tissues in unknown amounts.

Recently, there has been a resurgence of research into iron oxide materials for chemical/catalytic application [9,10,11,12,13,14,15]. Tartaj and co-workers [16] describe in their article entitled “The Iron Oxides Strike Back: …” how the exciting properties of iron oxides, coupled to their low toxicity, stability and economic viability, make them ideal for applications in a broad range of emerging fields. As one of the most significant earth oxides, iron oxide can be employed in the development of active and stable catalytic materials for reforming reactions to produce syngas [17,18,19,20,21], for production of chemicals [22,23,24], such as allyl alcohol [25,26], as an active component for catalytic oxidation in exhaust converters [27,28], for hydrodeoxygenation [29,30] and hydrogenation [31,32] reactions, for hydrogen sulfide removal from sewage [33], for electrochemical reduction of CO_2_ [34], in batteries [35], in chemical looping processes [36,37,38,39,40], in water gas shift reaction [41,42,43,44], etc. The use of iron in the proton exchange membrane (PEM) fuel cells [43,45] has also attracted special interest. Sebastian and co-workers [46] utilized Fe–N–C based catalyst as cathode in a direct methanol fuel cell (DMFC) in order to efficiently produce power. They reported an outstanding performance even at high methanol concentration, while at high temperature the catalyst displayed a similar current–time behavior to a membrane–electrode assembly based on a Pt cathode. Galvita and co-workers [47] suggested the use of iron-based materials for energy storage. Their concept includes a reactor configuration consisting of two chambers, both utilizing iron-based materials. Initially, the materials in the two chambers are reduced to metallic form, thus “charging” the reactor. In the second “discharging” step, steam is fed to the inner chamber, while air is sent to the outer. Hydrogen is produced by the inner chamber, whereas the external chamber is used for heat generation. Apart from iron, the external chamber contains a Ni-based layer, which is pyrophoric, in order to enable the startup of heat generation at room temperature under air flow.

In many of the aforementioned applications, the interest in iron is associated with the unique ability of the oxides to be reduced and then re-oxidized by H_2_O/CO_2_ [48]. Based on these iron oxide redox properties, a new reforming process has been developed by Buelens and co-workers [49], termed as “super-dry reforming”. The authors efficiently transformed CO_2_ from waste product to CO. They used Fe_2_O_3_ supported on MgAl_2_O_4_ as a solid oxygen carrier material (OCM), where three molecules of CO_2_ are consumed per one CH_4_, resulting in an enhanced CO production.

All of the above highlight the importance of iron/iron oxide systems, especially in the field of catalysis. Scientists consider iron-based materials as promising candidates to be employed in various chemical applications, like syngas production, chemical looping, methane decomposition for carbon nanotubes production and propane dehydrogenation. Therefore, this work focuses on reviewing the progress that has been made in the past few years, trying to unravel the role of iron in Fe-containing materials for sustainable application in chemical processes.

## 2. Fe in CeO_2_ for Chemical Looping

Chemical looping is a cyclic process where in the first half cycle, the materials undergo reduction through release of lattice oxygen producing e.g., CO, CO_2_ or H_2_O. In the second half cycle the oxygen vacancy in the lattice is refilled due to the reaction with an oxidizing gas, such as O_2_, CO_2_ or H_2_O, resulting in the production of CO or H_2_ [50]. Key properties for chemical looping are the reducibility of the carrier, its cost, toxicity, thermal stability and attrition resistance. Oxides of Ni, Cu, Mo, and Fe, are typically used as oxygen carriers [51,52]. Among these, iron oxides stand out because of their natural abundance and high reoxidation capacity with CO_2_ or H_2_O over a wide range of operating conditions (700–1000 °C). However, pure iron oxides tend to deactivate rapidly [53,54]. The major factor for deactivation in pure iron oxide materials is sintering. To overcome this challenge, iron oxides are often modified with other oxide materials, e.g., MgO, TiO_2_ [55], Al_2_O_3_ [56], CeO_2_ [57], ZrO_2_ [58,59], CeZrO_2_ [60], SiO_2_ [61], and MgAl_2_O_4_ [57,62,63,64]. Certain promoters contribute towards the redox reaction, alongwith iron oxide. These are therefore termed chemically active promoters, e.g., CeO_2_, CeZrO_2_ among the latter, CeO_2_ stands out as it has high activity toward methane oxidation by lattice oxygen, as well as reasonable H_2_O or CO_2_ reoxidation capacity [65].

**Figure 2:**
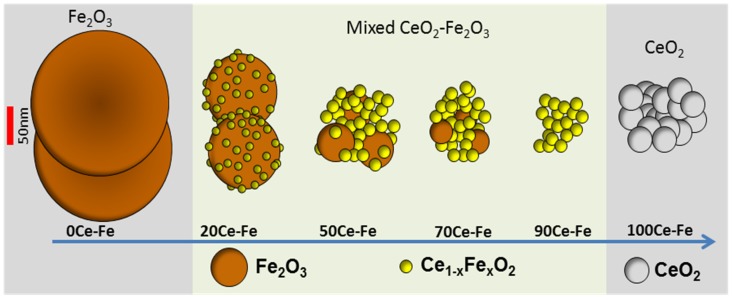
Schematic illustration of mixed CeO_2_–Fe_2_O_3_ samples, based upon ICP composition, XRD patterns, STEM, EDX, and EELS. Obtained from [65].

The interaction between Ce and Fe was found to induce structural modification and stabilization of iron oxides, making it an ideal candidate for promoting iron oxide in a chemical looping process. CeO_2_ improves the activity of Fe_2_O_3_ toward selective CH_4_ oxidation by lattice oxygen, as well as the re-oxidation capacity by H_2_O or CO_2_ [50,57,66]. The interaction can be established through the formation of a solid solution where Fe^3+^ cations dissolve in the ceria structure. The evolution of the Fe_2_O_3_–CeO_2_ structure as a function of composition is shown in Figure 2. In general, the formation of a solid solution between CeO_2_ and MeO_x_ (Me = Mn, Fe, or Cu) is responsible for enhancing the CeO_2_ reducibility compared with pure CeO_2_ [48,65,67]. CeO_2_ has a fluorite structure, with each Ce^4+^ cation surrounded by eight equivalent nearest O^2−^, that form the corners of a cube. When Ce^4+^ ions are replaced by lower valence cations, an oxygen vacancy or lattice defect can be created, which is considered to be the most reactive site. Both surface and bulk oxygen vacancies tend to form within CeO_2_, the former being suitable for adsorption purposes. 

Although the reduction at the surface of Fe_2_O_3_–CeO_2_ is independent of whether CeO_2_ is present or not, after consuming the available surface oxygen for CH_4_ oxidation, oxygen can be transferred from bulk to surface more rapidly in Fe_2_O_3_–CeO_2_ than in Fe_2_O_3_. This was ascribed to the CeO_2_ additive, creating oxygen vacancies in the solid solution. These vacancies are able to quickly transfer oxygen from the bulk to the surface of the oxygen carrier material through vacancy diffusion or even oxygen tunnels formed by vacancies. According to reported CH_4_-TPR profiles [50] for both CeO_2_ and Fe_2_O_3_–CeO_2_ samples, the removal of the most reactive oxygen mainly occurs at lower temperatures (773–823 K), giving rise to deep oxidation of CH_4_ to CO_2_, while CO is the main product at higher temperature (>873 K). Pure CeO_2_ shows a high CH_4_ oxidation activity at a temperature around 923 K due to the high consumption rate of surface lattice oxygen. In comparison to the profile of CeO_2_, Ce_0.9_Fe_0.1_O_2−*δ*_ shows a dramatic decrease of CO_2_ production with an increase in production of partial oxidation reaction products. The Fe_2_O_3_–CeO_2_ mixed oxides with Fe content above 0.5 fail to increase the conversion of CH_4_ and show a decline in CO selectivity, which is due to the increasing amount of pure Fe yielding deep oxidation products. Therefore, an equal weight loading of Fe and Ce can maximally promote the reactivity for redox reactions of the material [48,50]. 

Overall, three types of deactivation were identified for the Fe_2_O_3_–CeO_2_ materials: (1) Fe extraction from the solid solution Ce_1−x_Fe_x_O_2_, (2) perovskite formation (CeFeO_3_) and (3) sintering. The extraction of Fe from the Ce_1−x_Fe_x_O_2−x_ occurs very fast. It leads to lower reducibility of CeO_2_, but at the same time provides more iron oxide storage capacity by setting free extra Fe. CeFeO_3_ perovskite formation leads to loss of oxygen storage capacity as it is non-reducible at temperatures lower than 1073 K. Finally, sintering is a slow process which continues throughout cyclic operation. It causes crystallites to grow in size, thereby increasing the diffusion time of bulk oxygen to the surface. Hence, a lower degree of reduction is reached in a given reduction time and upon re-oxidation with CO_2_, a lower CO yield is obtained. The relative importance of these deactivation types depends on the composition of the oxygen storage materials. In iron rich samples deactivation is predominantly caused by sintering of iron oxides. Fe extraction is of minor importance given the composition of this material. Similarly, perovskite formation may occur, but will hardly affect the cycling productivity. In ceria rich samples, all three types of deactivation occur. Compared to pure Fe_2_O_3_, sintering as the main deactivation type is tempered by the strategy of decorating Fe_2_O_3_ with CeO_2_ nanoparticles. 

## 3. Fe in Spinels for Chemical Looping

One of the most common iron containing chemical compounds with spinel structure is Fe_3_O_4_. It naturally occurs as the mineral magnetite, containing Fe^2+^ and Fe^3+^ ions. Nano-Fe_3_O_4_ has recently gained attention as heterogeneous catalyst due to its environmental compatibility, simple handling and ease of recovery using an external magnetic field [68,69,70]. There are many reports in literature using Fe_3_O_4_-based materials in environmental applications [69,71], in Fenton-like processes [68,72] and in wastewater treatment [9,73,74].

**Figure 3:**
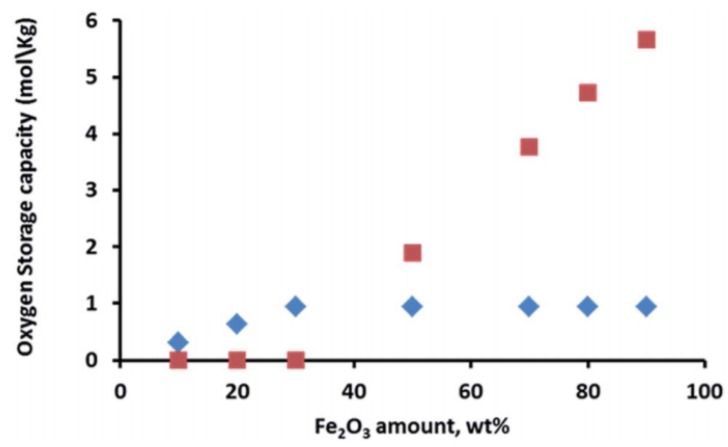
Oxygen storage capacity of MgFe_x_Al_2−x_O_4_ materials as a function of the Fe_2_O_3_ content. Note that when Fe_2_O_3_ loading is less than 30 wt %, it is completely incorporated into the spinel structure without separate Fe_2_O_3_ phases. 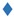: iron incorporated in spinel structure; 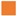: separate Fe_2_O_3_ phase. Obtained from [81].

Iron can also form spinel phases with aluminum and magnesium, depending on the applied conditions during the catalyst synthesis, e.g., calcination temperature, resulting in FeAl_2_O_4_ and MgFe_2_O_4_ structures, respectively [75,76]. These materials have been used as oxygen storage during chemical looping processes, preventing the sintering of Fe particles and thus increasing the process stability. Ferrites have also been utilized for oxidation of alcohols to the corresponding ketones or aldehydes [77,78]. However, the aforementioned iron spinel structures require higher reduction/oxidation temperature, resulting in more severe operating conditions [79,80]. On the other hand, Dharanipragada and co-workers [81] synthesized a novel material, combining Al^3+^, Fe^3+^ and Mg^2+^ in one spinel structure, forming a MgFe_x_Al_2−x_O_4_ material that was used for oxygen storage during chemical looping for CO_2_ to CO conversion. They concluded that at low Fe loading (<30 wt %), most of the iron is in a spinel structure with magnesium aluminate. Even though Fe incorporated inside the spinel has lower oxygen storage capacity compared to Fe_2_O_3_ supported on the MgFe_x_Al_2−x_O_4_ material (Figure 3), the stabilization of Fe in the spinel structure results in an improved performance.

**Figure 4:**
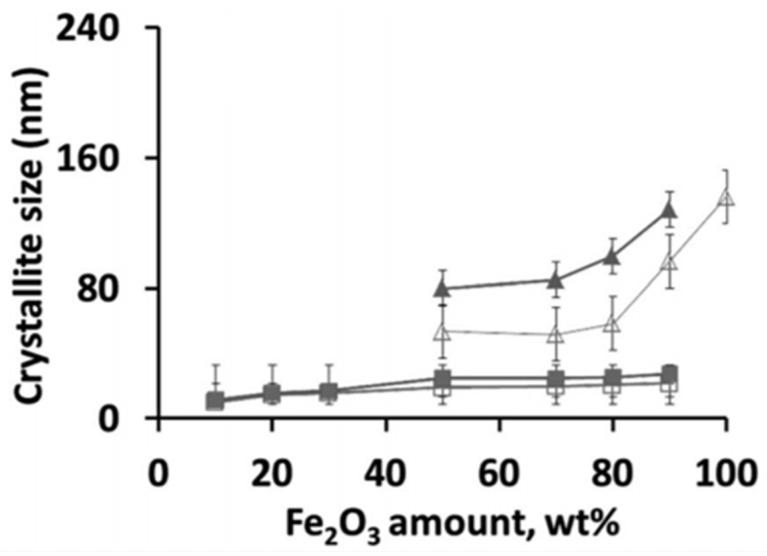
Crystallite size of Fe_2_O_3_ and MgFe_x_Al_2−x_O_4_ phases in the samples, as calculated based on XRD using the Scherrer equation. As-prepared: (□) MgFe_x_Al_2−x_O_4_ and (Δ) Fe_2_O_3_; (■) MgFe_x_Al_2−x_O_4_ and (▲) Fe_3_O_4_ after 5 isothermal redox cycles of H_2_/CO_2_ at 1023 K. Obtained from [81].

The occurrence of a separate Fe_2_O_3_ phase will drastically increase the oxygen storage capacity of the material. When Fe is fully incorporated into the spinel, redox cycling proceeds between Fe^3+^ and Fe^2+^, based on Mossbauer spectra [81]. On the other hand, for the materials with higher Fe_2_O_3_ loadings, the cycling of MgFe_x_Al_2−x_O_4_ + Fe_2_O_3_ will change the Fe oxidation state between Fe^3+^ and Fe^2+^ in the spinel and between Fe^3+^ and Fe^0^ in the separate iron oxides. However, the latter materials do suffer from severe sintering. Figure 4 shows that already after five isothermal cycles under H_2_/CO_2_ at 1023 K, the crystallite size for Fe_2_O_3_ in MgFe_x_Al_2−x_O_4_ with 50 wt % Fe_2_O_3_ increased from 60 to 80 nm, while the size of the MgFe_x_Al_2−x_O_4_ remained stable at 10–22 nm [81]. This implies that the incorporation of Fe inside the lattice of the magnesium aluminate spinel structure can greatly improve the stability of the material during chemical looping, alternating between reducing and oxidizing environment. And this stability of the material determines the economics of the process [48,82,83,84].

**Figure 5:**
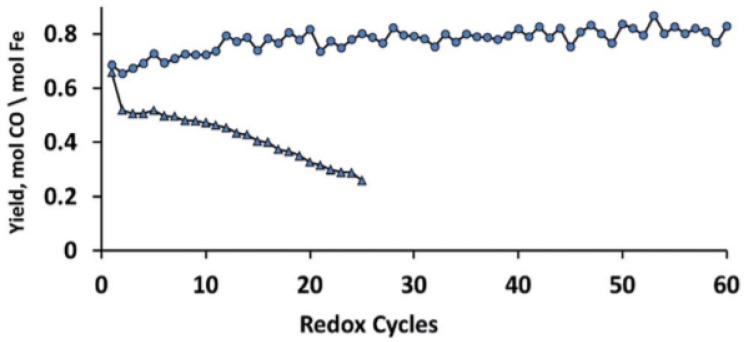
CO yield in CO_2_ to CO conversion as a function of isothermal H_2_-CO_2_ redox cycles for MgFe_x_Al_2−x_O_4_ and Fe_2_O_3_/MgFe_x_Al_2−x_O_4_ with (●) 10 wt % Fe_2_O_3_ (MgFe_0.14_Al_1.86_O_4_) and (▲) 90 wt % Fe_2_O_3_. Each cycle (16 min) is composed of 4 min H_2_ (5% in Ar), 4 min He, 4 min CO_2_ (100%) and 4 min He at 1123 K. All the gas flows were 1.1 NmL/s. Obtained from [81].

The MgFe_0.14_Al_1.86_O_4_ spinel structure with 10 wt % Fe_2_O_3_ (x = 0.14) shows the highest stability during isothermal H_2_/CO_2_ cycles without any Fe_2_O_3_ phase segregation (Figure 5). Dharanipragada and co-workers [85] further examined the reduction kinetics of this MgFe_0.14_Al_1.86_O_4_ using XRD and in-situ QXANES at the Fe–K edge. They found that Fe is incorporated in the octahedral sites of the spinel, replacing Al in the lattice.

**Figure 6:**
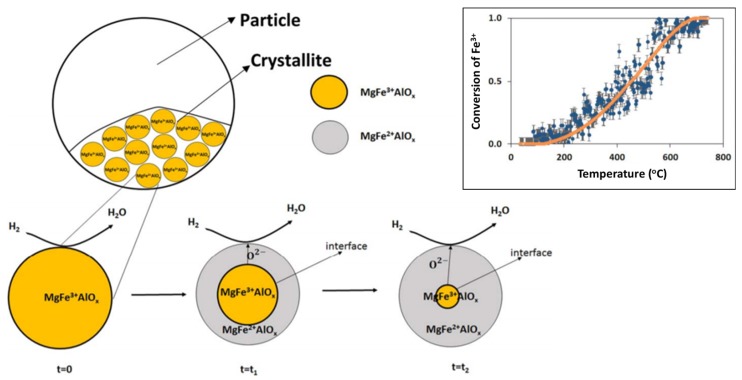
Schematic representation of the shrinking core model in a MgFe_x_Al_2−x_O_4_ crystallite. Top right inset: Observed and calculated conversion profile of Fe^3+^ based on pre-edge fitting of QXANES spectra from MgFe_0.14_Al_1.86_O_4_. Obtained from [85].

During reduction, 55% of Fe could be reduced from 3+ to 2+, with the rest remaining identical to the “as-prepared” state. A shrinking core model was proposed [85], where initially the external surface of the solid is involved in the reaction (reduction). The reduced layer then thickens, depending on the exposure time under reducing environment, enclosing a shrinking core of unreacted solid (Figure 6). This shrinking core model provided an adequate description for the transition from Fe^3+^ to Fe^2+^ in the MgFe_0.14_Al_1.86_O_4_ material (top right inset of Figure 6).

On the crystallite scale, the solid-solid transformations are governed by three phenomena according to Dharanipragada and co-workers [85]: (1) Reaction of surface oxygen with H_2_, forming H_2_O, (2) reduction of MgFe_x_^3+^Al_2−x_O_4_ to MgFe_x_^2+^Al_2−x_O_4_ at the interface between unreacted core of the crystallite and reduced material and (3) oxygen diffusion from the core through the reduced layer to the surface, where the reaction takes place.

Fe-based spinel materials and more specifically the MgFe_x_Al_2−x_O_4_, are recently receiving more attention as they combine good redox properties and thermal stability. They can be applied to many processes varying from pollutants removal, e.g., SO_2_ [86] and chemical looping [81] to syngas production via catalytic steam and/or dry reforming.

## 4. Fe in Nano-Alloys for Catalysis

Many applications of iron use it in alloyed form, steel being the most famous Fe-containing alloy. In catalysis, Fe-containing nano-alloys are often used, e.g., in bimetallic nano-alloys combined with a noble metal or non-noble element. Yfanti and co-workers [70] used Fe–Pt catalysts for hydrodeoxygenation of glycerol. They reported an electronic interaction between Fe and Pt, which increased the glycerol conversion, compared to the monometallic Pt. By increasing the Fe content, the catalyst surface structure was changed, as the iron oxide clusters started to cover the Pt particles. This resulted in a slight decrease in the main product selectivity, 1,2-propanediol, but at the same time, the stability of the catalyst was increased. The improved catalytic performance was attributed to the Fe addition as it enhanced the carbon-resistance of the catalyst and prevented the sintering of Pt particles. Saravanan and co-workers [87] used Fe–Pt catalysts for the oxidation of indoor pollutants, such as CO and benzene, in a temperature range of 298–473 K, demonstrating that they can be a possible alternative for the existing monometallic Pt catalysts. The authors concluded that the intermetallic phase PtFe_3_ is more active than the Pt_3_Fe. On the other hand, Jiang and co-workers [88] used Fe–Pd bimetallic catalysts with a core-shell structure for the oxygen reduction reaction (ORR) as an alternative to Pt-based catalysts. They demonstrated that Fe–Pd had a robust catalytic activity and durability in ORR.

There is a number of studies in literature indicating the promoting effect of Fe to Rh-based catalysts for syngas conversion to C_2+_ oxygenates, such as ethanol [23,24,89,90,91,92]. An alloy based on Fe and Rh has been reported by Palomino and co-workers [91], who investigated the effect of alloying on syngas conversion. They found that the addition of Fe increased the selectivity towards ethanol, but partially suppressed the catalytic activity due to blocking or modifying of Rh active sites depending on the Fe content. Similarly, Liu and co-workers [93] used Rh supported on SiO_2_ catalysts promoted with Mn and Fe for CO hydrogenation towards light hydrocarbons and oxygenates. A trimetallic Rh-Fe-Mn alloy was formed, with molar ratio of 1:0.15:0.10, that resulted in higher selectivities than the bimetallic counterparts.

**Figure 7:**
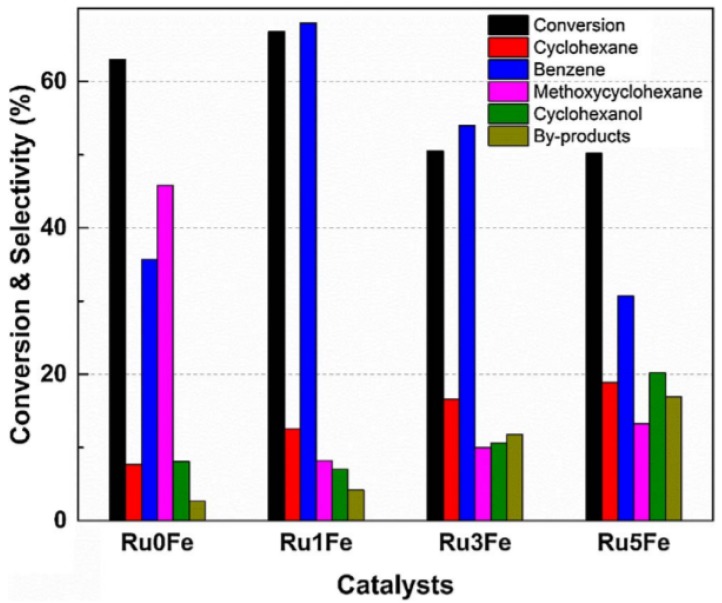
Conversion and product distribution of the Ru_x_Fe/TiO_2_ catalysts, during anisole hydrodeoxygenation (HDO), in a stainless-steel batch reactor. Reaction conditions: 10 wt % anisole (40 mL), catalyst (1.0 g), 200 °C and 10 bar H_2_ for 3 h. Obtained from [94].

A synergetic effect of Fe and Ru supported on TiO_2_ was reported by Phan and co-workers [94] during anisole hydrodeoxygenation reaction (HDO). The addition of Fe to the Ru/TiO_2_ catalyst altered the surface properties, changing the reaction pathway. More specifically, the anisole conversion and product distribution were affected by the Fe loading (Figure 7). The combination of Ru and Fe lead to a higher selectivity of benzene and a lower selectivity of methoxycyclohexane, indicating that direct deoxygenation (DDO) is the main reaction pathway. The enhanced performance with Fe was attributed to the increased number of oxygen vacancies on the surface of the TiO_2_ support.

Tungsten forms alloy with Fe and retains functional properties (mechanical, magnetic, etc.) even at elevated temperature, having a variety of applications in the industrial sector [95]. Tharamani and co-workers used the Fe–W alloy as an anode in a methanol oxidative fuel cell with a H_2_SO_4_ medium [95]. Shi and co-workers [96] used Cu–Fe bimetallic catalysts supported on carbon nanotubes for the synthesis of higher alcohols from syngas. They found that the selectivity toward methanol decreased, and the formation of C_2+_–OH alcohols increased, reaching a selectivity of 68.8% for the best candidate with a Fe:Cu atomic ratio of 1. A Fe–Co alloy phase was reported to form after reduction in hydrogen by Koike and co-workers [97]. This Fe–Co catalyst was active for toluene steam reforming, but deactivated due to oxidation of the alloy phase. The addition of hydrogen in the feed stream resulted in higher activity.

In what follows, the bimetallic Ni-containing Fe nano-alloys will be discussed in detail as they have an outstanding ability to limit surface carbon accumulation.

### 4.1. Fe–Ni Nano-Alloy

**Figure 8:**
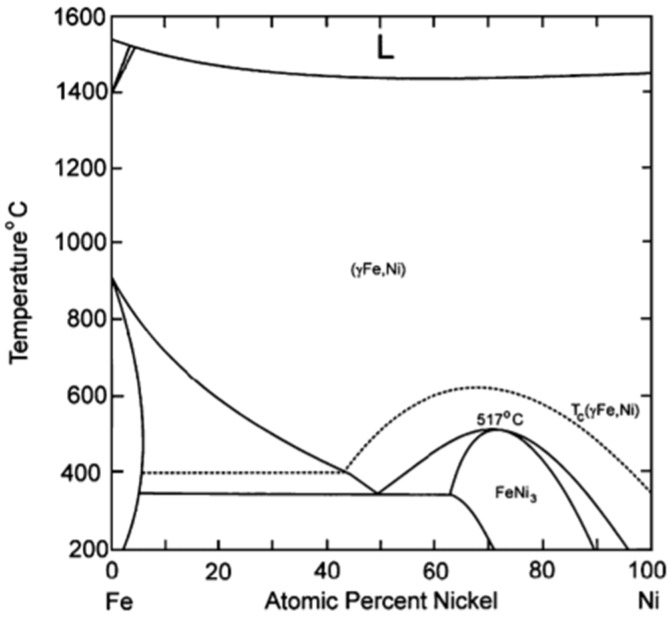
Phase diagram of the bimetallic Fe–Ni system. Obtained from [99].

The preparation of a Fe–Ni alloy generally involves impregnation of their precursors on a support material, calcination under air and reduction [20,98]. However, this might result in large and non-uniform Fe–Ni particles [20]. According to the Fe–Ni phase diagram (Figure 8) [99], at least one regular Ni-rich alloy with FeNi_3_ composition is known. Other Fe–Ni alloy structures with composition NiFe, Ni_3_Fe_2_ and Ni_2_Fe have also been reported [100]. However, a bimetallic Fe–Ni system will most likely contain a wide range of different structures of the nano-alloy, depending on the Fe/Ni ratio and the applied temperature. Figure 8 shows that Ni and Fe, as well as their alloys, have similar melting points. This implies that the surface migration and aggregation phenomena, which are correlated with the Tammann temperature (=0.52·melting point), will be within the same temperature range. 

Co-impregnation was used by Theofanidis and co-workers [17] to prepare Fe–Ni catalysts supported on MgAl_2_O_4_. A surface area of 84.7 ± 5.8 and 47.6 ± 11.4 m^2^·g^−1^ was measured for 8 wt %Ni-5 wt %Fe and 8 wt %Ni-8 wt %Fe (Ni/(Ni + Fe) ratios of 0.6 and 0.5), respectively, after the calcination step under air flow (named as “as-prepared”). Similar values, in the range of 53–71 m^2^·g^−1^, were obtained by Kustov and co-workers for Fe–Ni catalysts with different total metal loading and a Ni/(Ni + Fe) ratio varying from 0 to 0.8, supported on MgAl_2_O_4_ [100]. On the other hand, a Fe–Ni catalyst supported on Mg_x_Al_y_O_z_ hydrotalcite has been reported to have higher surface area, in the range of 172–175 m^2^·g^−1^ [101]. Li and co-workers [19] also prepared Fe–Ni, as a steam reforming catalyst, using a hydrotalcite type of precursor. They obtained uniform Fe–Ni nanoparticles, with particle size varying from 8.1 to 10.2 nm depending on the Ni/(Ni + Fe) ratio (from 0.4 to 0.9).

**Figure 9:**
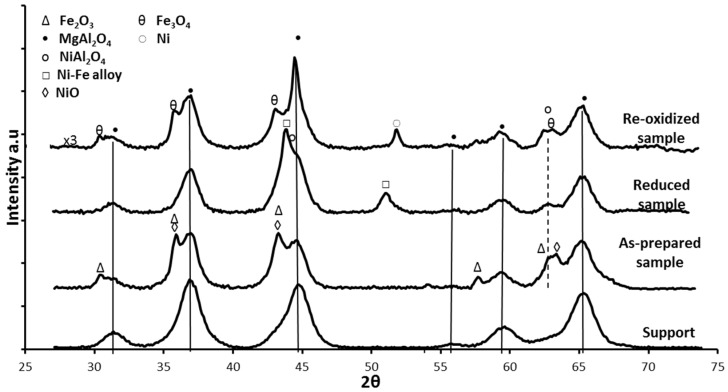
Full XRD scans of MgAl_2_O_4_, as-prepared, reduced and re-oxidized 8 wt %Ni-5 wt %Fe/MgAl_2_O_4_ (1 mL/s of 10%H_2_/He mixture or CO_2_ at a total pressure of 101.3 kPa and 1123 K). The NiFe_2_O_4_ phase cannot be distinguished due to overlapping with Fe_2_O_3_. Reproduced from [17].

The crystalline phases of the Fe-Ni/MgAl_2_O_4_ samples were determined by X-ray diffraction (XRD). In the “as-prepared” state, NiO, NiAl_2_O_4_, NiFe_2_O_4_ and Fe oxides were detected, depending on the used support material [17,18,101]. Upon reduction, a bimetallic Fe–Ni nano-alloy with a crystallite size of approximately 5–20 nm is formed (Figure 9), depending on the metal (Ni and Fe) loading, shifting the main 2θ angle position to lower values than for metallic Ni [18,21,101]. The XRD pattern after oxidation by CO_2_ (Figure 9) shows that the Fe–Ni alloy was decomposed to Ni and Fe_3_O_4_, while the NiAl_2_O_4_ and MgAl_2_O_4_ support diffractions remained stable.

**Figure 10:**
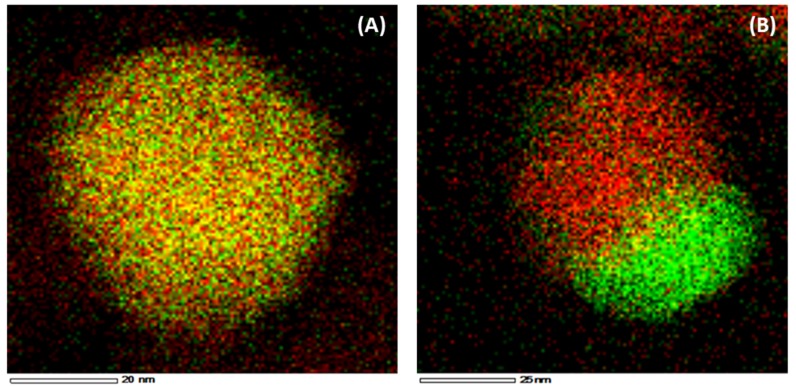
EDX element mapping of 8 wt %Ni-5 wt %Fe/MgAl_2_O_4_. (**A**) After H_2_-reduction (1 mL/s of 5%H_2_/Ar mixture at a total pressure of 101.3 kPa and 1123 K). (**B**) After CO_2_ oxidation (1 mL/s of CO_2_ at a total pressure of 101.3 kPa and 1123 K). Red and green colors correspond to Fe and Ni elements respectively. Obtained from [17].

The Ni and Fe elements are uniformly distributed in the nano-alloy (Figure 10A) after reduction. In contrast, after CO_2_ oxidation Ni and Fe particles are segregated (Figure 10B) and Fe is oxidized to Fe_3_O_4_ [17,22,102].

**Figure 11:**
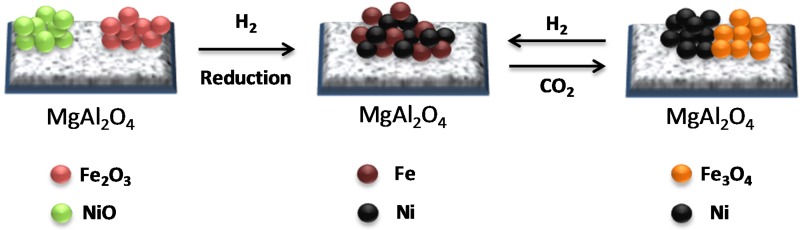
Schematic diagram of Fe–Ni nano-alloy formation and decomposition, depending on the applied environment. Obtained from [17].

A schematic illustration of the Fe–Ni nano-alloy formation and decomposition is presented in Figure 11. The alloy is decomposed during CO_2_ oxidation between 850 K and 1123 K yielding two separate phases of Ni and Fe_3_O_4_ (see the EDX elemental mapping image Figure 10B). Metallic Ni in the bulk cannot be oxidized to NiO under CO_2_ flow up to 1123 K. A subsequent H_2_ reduction step leads again to the formation of a Fe–Ni nano-alloy [17]. 

#### 4.1.1. Activity during Methane Decomposition

Monometallic [103,104] and bimetallic Fe-based catalysts were extensively used for carbon formation [105,106,107]. Even if the carbon formation and growth on catalysts is an undesired phenomenon in reforming reactions, the synthesis of carbon nanotubes (CNT), a type of carbon material with graphite layers and tubular structure, plays a very important role in the field of nanotechnology [105,106,107]. Carbon nanotubes were first identified by Lijma [108]. They require a source of elemental carbon, such as methane, and energy in order to be formed. The CNTs have numerous properties like high surface area, electronic and thermal conductivity, tensile strength, resistance to acidic/basic chemicals, making them ideal to be used in a variety of applications such as catalyst supports, air and water filtration, conductive adhesive, fibers and fabrics, etc. [109].

**Figure 12:**
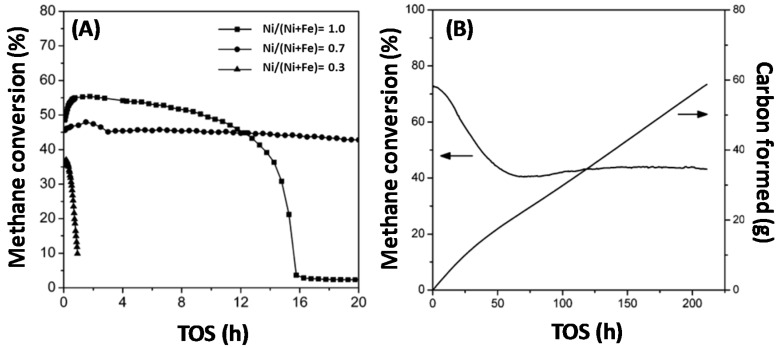
(**A**) Methane conversion over Fe–Ni catalysts with Ni/(Ni + Fe) ratio of 1.0, 0.7 and 0.3 as a function of TOS at 873 K. (**B**) Long term test of a Fe–Ni catalyst with Ni/(Ni + Fe) ratio of 0.7 during methane decomposition at 923 K. Reproduced from [110].

**Figure 13:**
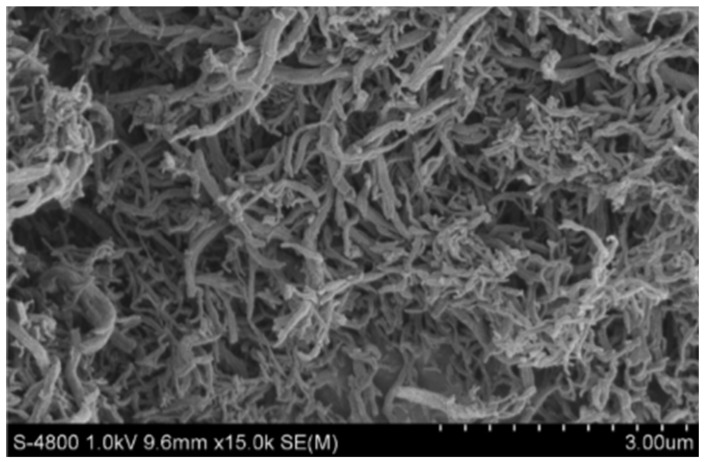
SEM image of carbon nanotubes produced over a Fe–Ni catalyst with Ni/(Ni + Fe) of 0.7 after 210 h TOS under methane decomposition at 923 K. Obtained from [110].

Methane is often used as a carbon source and the understanding of its activation step, which typically occurs over metals, is essential. The activation of CH_4_ only, without co-feed of other reagents, under methane decomposition (MD) reaction conditions, at 1023 K and 1 bar under the flow of 1 mL/s 50%CH_4_-50%Ar, over monometallic Ni, Fe and bimetallic Fe–Ni, was investigated by Theofanidis and co-workers [17]. Carbon accumulated according to the methane decomposition reaction (CH_4_→ C + 2H_2_) [102]. After oxidation by CO_2_, it was found that more carbon was deposited on the bimetallic catalyst than on the monometallic ones, implying that the Fe–Ni alloy does not suppress carbon formation. Wang and co-workers used Fe–Ni catalysts with different Ni/(Ni + Fe) ratios for methane decomposition (Figure 12) in order to produce hydrogen and carbon nanotubes (Figure 13) [110]. They also found that the Fe–Ni alloy is active for methane decomposition. Figure 12A shows the methane conversion as a function of time-on-stream (TOS) for three catalysts with Ni/(Ni + Fe) ratio of 1.0, 0.7 and 0.3 respectively. The monometallic Ni (Ni/(Ni + Fe) of 1.0) deactivated after 16 h TOS, while the Fe-rich sample (Ni/(Ni + Fe) of 0.3) displayed almost no activity, as it was completely deactivated after less than 2 h TOS. On the other hand, the bimetallic Fe–Ni catalyst with a Ni/(Ni + Fe) ratio of 0.7 had a stable performance throughout 20 h TOS. They further examined the best candidate for the same reaction for longer TOS (Figure 12B). The conversion dropped from 72% to 40% in the first 50 h, while hereafter the catalyst remained stable, even up to 210 h TOS. 56.2 g of carbon were produced, Figure 12B, which equals 562 g of C/g of catalyst during the 210 h. 

According to many researchers, the carbon accumulation follows the deposition-diffusion-precipitation mechanism (or bulk diffusion mechanism) [110,111,112,113], where the properties of the metal play a crucial role. The modification of the Ni catalyst with Fe may increase the carbon diffusion rate, thereby decreasing the surface carbon accumulation. Indeed, the diffusion of carbon atoms in Fe is 3 orders of magnitude faster than in Ni [114]. The fast removal of carbon atoms from the surface can suppress the reverse reaction of methane formation (C + 2H_2_→ CH_4_), thus compensating for the lower methane decomposition rate of bimetallic Fe–Ni catalysts compared to monometallic Ni. Indeed, Ni is more active than Fe for methane decomposition and hence the addition of Fe is likely to reduce the carbon formation rate. As a result, the balance among carbon formation, diffusion and precipitation as carbon nanotube is maintained in Fe–Ni catalysts leading to improved catalytic performance [110].

#### 4.1.2. Activity during Syngas Production

**Figure 14:**
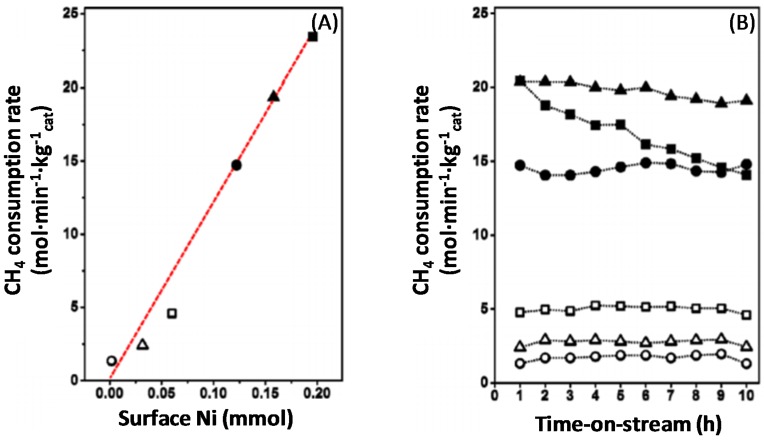
(**A**) Rate of methane consumption (mol·min^−1^·kg^−1^_cat_) as a function of the amount of surface Ni (mmol), during methane dry reforming. ■: Pure Ni; ▲: Ni/(Ni + Fe) = 0.80; ●: Ni/(Ni + Fe) = 0.75; □: Ni/(Ni + Fe) = 0.5; ∆: Ni/(Ni + Fe) = 0.25 and ○: Pure Fe, all supported on Mg_x_Al_y_O_z_ and (**B**) rate of methane consumption (mol·min^−1^·kg^−1^_cat_) as a function of time-on-stream (TOS) during DRM at 923 K. Reproduced from [101].

Syngas production over Fe–Ni catalysts strongly depends on the composition of the nano-alloy that is formed after the reduction process [17,21,101]. More specifically, the Fe–Ni catalysts are sensitive to the Fe content and their activity is related to the employed Ni/Fe [17,21] or Ni/(Ni + Fe) ratio (Figure 14A) [101].Wang and co-workers [21] found that the addition of Fe promoted the steam reforming reaction in the range of Ni/Fe ≥ 2. On the other hand, Theofanidis and co-workers [17] found a slight improvement in the activity of Ni-Fe catalysts in the same range of Ni/Fe ratio, while the carbon deposition was suppressed remarkably. Pure Fe is twenty times less active than a pure Ni catalyst for methane dry reforming (DRM) at 923 K, Figure 14B, with a CH_4_ consumption rate of 0.022 mol·s^−1^·kg^−1^_cat_ and 0.34 mol·s^−1^·kg^−1^_cat_, respectively. However, pure Ni loses 30% of its activity after only 10 h TOS. On the other hand, the bimetallic Ni-rich Fe catalysts, with Ni/(Ni + Fe) ratios of 0.8 and 0.75 show an activity similar to pure Ni at 923 K, 0.32 and 0.25 mol·s^−1^·kg^−1^_cat_, respectively. Their stable performance is emphasized by their modest activity loss during 10 h TOS, by only 6.4% and 4.0%, respectively [101]. 

**Figure 15:**
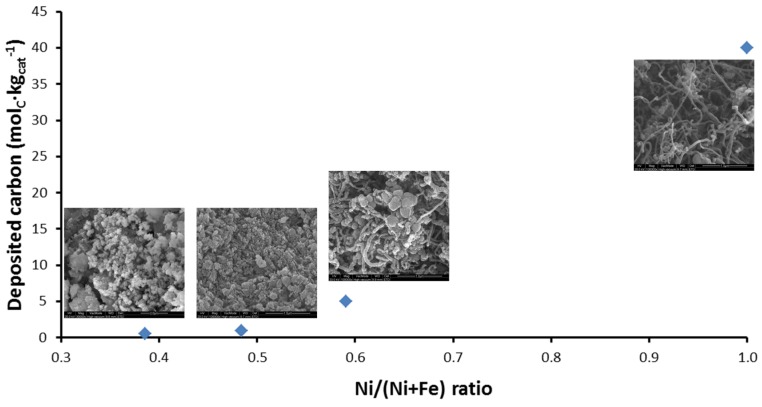
Deposited carbon as a function of Ni/(Ni + Fe) ratio along with the SEM micrographs of “spent” catalysts supported on MgAl_2_O_4_. Temperature 1023 K, CH_4_:CO_2_ = 1:1, reaction time 4 h. Reproduced from [17].

The deposited carbon as a function of Ni/(Ni + Fe) ratio can be seen in Figure 15. Carbon filaments start to grow as the Ni/(Ni + Fe) ratio approaches 1 (pure Ni) [115] after 4 h TOS. On the other hand, a negligible amount of carbon was accumulated on bimetallic Fe–Ni with Ni/(Ni + Fe) ratio ≤ 0.6 (Figure 15).

**Figure 16:**
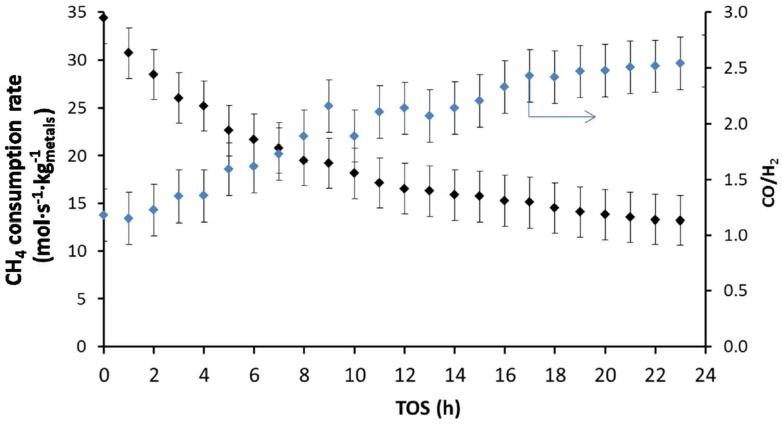
CH_4_ consumption rate (mol_CH4_·s^−1^·kg^−1^_metals_) and the produced CO/H_2_ ratio over a bimetallic Fe–Ni catalyst with Ni/(Ni + Fe) = 0.65 during DRM at 1023 K (total pressure of 101.3 kPa and CH_4_:CO_2_ = 1:1). W_metals_/F^0^_CH4_ = 0.025 mol_CH4_·s^−1^·kg^−1^_metals_, X_CH4_: from 62% to 24%. Reproduced from [116].

During a stability test over longer time-on-stream for DRM at 1023 K (Figure 16), Theofanidis and co-workers [116] observed a loss of 62% in the CH_4_ consumption rate of a bimetallic Fe–Ni catalyst supported on MgAl_2_O_4_ with Ni/(Ni + Fe) of 0.65. They examined carbon formation as a possible reason for the deactivation. However, the deposited carbon was below detection limits after 24 h TOS, implying that the addition of Fe increased the carbon-resistance of the catalyst during reforming reactions. They also evaluated the reversibility of the observed deactivation. As much as 76% of the catalyst initial activity could be restored [116]. Since no carbon was deposited, it was concluded that sintering was at the origin of the irreversible deactivation that accounted for the persisting 24% of activity loss. The reversible deactivation was attributed to Fe segregation from the Fe–Ni nano-alloy structure. Indeed, an increase in CO/H_2_ ratio from 1.3 after 1 h TOS to 2.5 after 24 h TOS (Figure 16) was observed, indicating a modification in the nature of active sites during the reaction. As Fe is more active for the reverse water-gas-shift reaction (RWGS: ${\mathrm{CO}}_{2}+H_{2\;}{\;H}_{2}O+\mathrm{CO}$) than Ni, its segregation from the alloy leads to consumption of H_2_ and hence an increase in CO/H_2_ ratio. The Fe–Ni nano-alloy can however be reconstructed upon regeneration and reduction steps.

**Figure 17:**
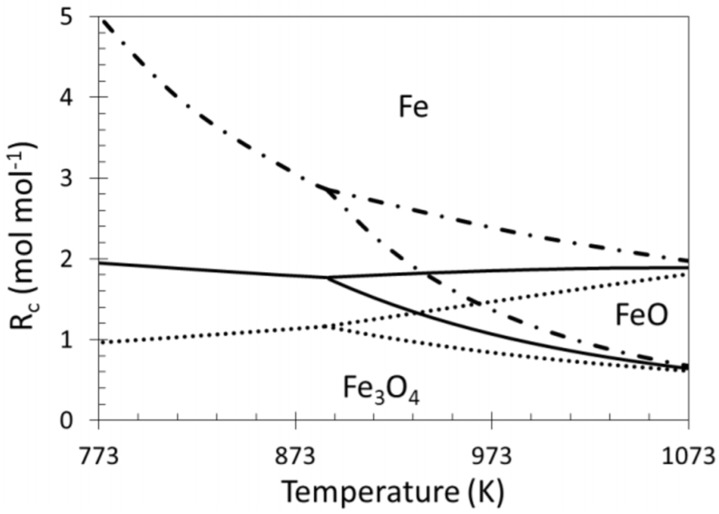
Phase diagram showing the equilibrium lines between Fe_3_O_4_, FeO and Fe as a function of temperature and reduction capacity in presence of: 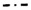 H_2_/H_2_O; 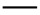 H_2_/CO/H_2_O/CO_2_ (equimolar amount of C and H_2_ corresponding with a feed of CH_4_ + CO_2_); 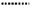 CO/CO_2_. Obtained from [49].

The ratio between reducing and oxidizing gases determines the material’s position in the iron/iron oxides system and is as such very important for the stability of Fe containing alloys (Figure 17) [22,49,117]. The outlet gas of a reforming reaction contains syngas, a mixture of CO and H_2_, both reducing gases, as well as unreacted CO_2_ and H_2_O, from the reverse water-gas-shift reaction, as oxidizing gases. The reduction potential of this gas mixture strongly depends on the ratio between reducing and oxidizing gases. Indeed, the presence of CO_2_ or H_2_O in the reaction mixture significantly decreases the achieved reduction degree of iron oxide because they both act as oxidizing agents. The ratio *R_c_*, or reduction capacity, which indicates the reducing strength of the gas composition, can be expressed as follows:

*R_c_* = (CO + H_2_)/(CO_2_ + H_2_O) (1)

**Figure 18:**
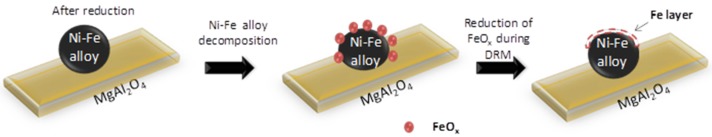
Deactivation due to Fe segregation from the Fe–Ni surface alloy during DRM at high temperature (1023 K). Obtained from [116].

However, during methane reforming, iron involved in the CO_2_ or H_2_O activation will be segregated from the Fe–Ni alloy [118], even under an overall reducing environment (*R_c_* > 1). This redistribution of elements could eventually result in Fe species located on top of alloy particles [101,116]. Wang and co-workers examined Fe–Ni catalysts supported on Al_2_O_3_ for steam reforming of tars and used Extended X-ray absorption fine structure (EXAFS) spectroscopy to analyze the local structure of the Fe–Ni nano-alloys [21]. They found a lower coordination number for Fe than for Ni, suggesting that Fe/Fe oxide species are enriched in the outer layers of the alloy particles. These iron species can further interact with the C, CH_x_ and H species at the surface. A similar mechanism of deactivation can be invoked for any high concentration Fe containing alloy: It can decompose at high temperature under H_2_O/CO_2_ [17,22,102], resulting in segregation of Fe from the alloy (Figure 18). The deactivation can then be attributed to the lowered surface Ni/Fe ratio, since Fe is less active in reforming than Ni [17,101]. All of the above implies that even if *R_c_* can determine the oxidation state of Fe under reaction conditions, the local interaction of Fe with oxidizing gases will lead to iron segregation, independent from the reduction capacity *R_c_*.

Theofanidis and co-workers [116] evaluated the thermodynamic tendency of Fe to move towards the alloy surface using Density Functional Theory. They compared this tendency of Fe in a bimetallic Fe–Ni and a trimetallic catalyst, containing a noble metal, Pd, Fe–Ni–Pd (Table 1). The DFT calculations reveal that (i) the segregation behavior of Fe is a very strong function of the adsorbate layer present, and (ii) the presence of Pd in a Fe–Ni alloy will reduce the tendency of Fe to segregate to the surface for coverages that are close to what can be expected during DRM conditions.

#### 4.1.3. Catalyst Regeneration: Carbon Removal by CO_2_

Despite the different ways to control catalyst deactivation due to carbon deposition, carbon accumulation will eventually occur during reforming reactions and thus regeneration will be required in order to remove all carbon species [119,120]. Therefore, it is important to understand the catalyst regeneration mechanisms. The rate of carbon removal depends on its structure [121], location [122] and on the nature of the catalyst [123,124,125].

The existence of two different carbon species structures, graphitic and amorphous, was observed by Guo and co-workers [126], who performed Raman spectroscopy over Ni/MgAl_2_O_4_ after coking via CH_4_ temperature programmed decomposition. Raman spectroscopy is widely used in order to investigate the structure and crystallite size of carbon species [127]. It provides information about the electronic properties and can detect the presence of ordered carbon species [126]. The Raman spectrum of a single crystal graphene sample only shows the G band at approximately 1581 cm^−1^ Raman shift. However, in case of imperfect, polycrystalline graphite and other carbonaceous materials [128], additional bands are detected at 1355 cm^−1^ (D band) and 1620 cm^−1^ (D’ band). The ratio of areas I_D_/I_G_ has been correlated to the inverse crystallite size of graphite [129]. 

**Figure 19:**
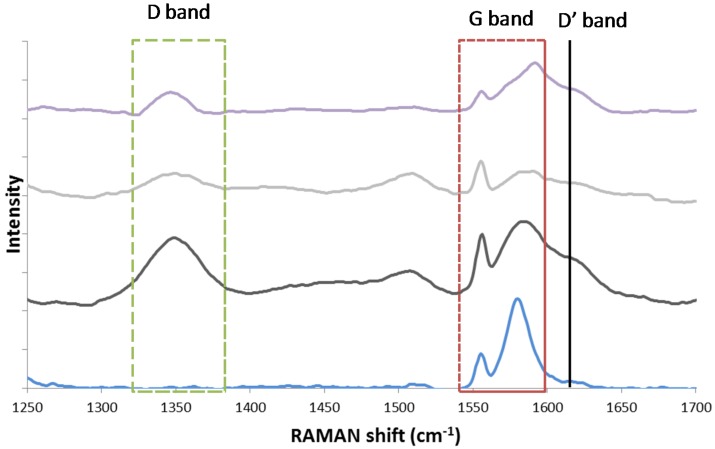
Raman spectrum of the spent Fe–Ni catalyst, with Ni/(Ni + Fe) ratio of 0.6 (DRM for 1 h, 1023 K, CH_4_:CO_2_ = 1.1, total pressure of 101.3 kPa). Blue line: pure graphite as a reference, black line: spent Fe–Ni catalyst, grey line: spent Fe–Ni catalyst after CO_2_-TPO up to 950 K, purple line: Spent Fe–Ni catalyst after CO_2_-TPO up to 1123 K. Obtained from [118].

**Figure 20:**
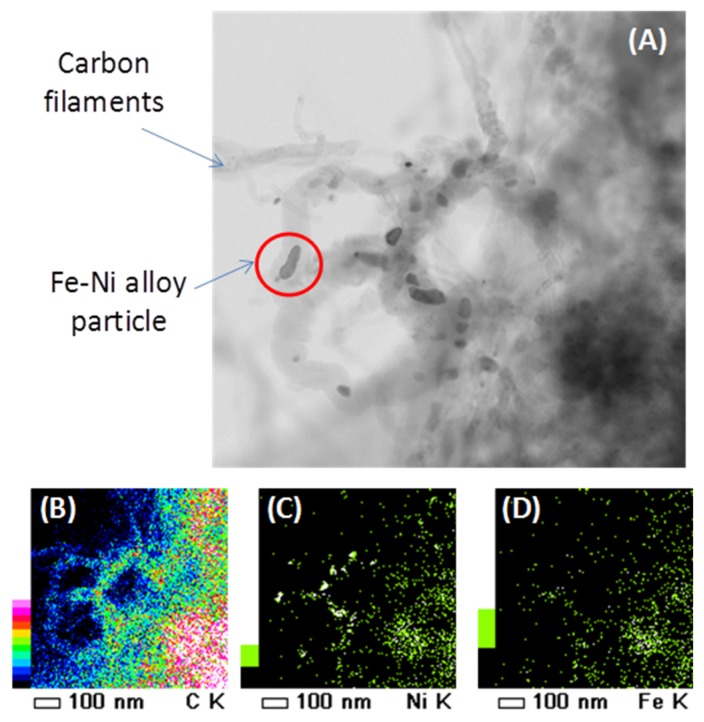
(**A**) HRTEM image of a spent Fe–Ni catalyst with Ni/(Ni + Fe) ratio of 0.6 (after DRM at 1023 K, CH_4_:CO_2_:He = 1.1:1:1, total pressure of 101.3 kPa, reaction time 1 h). EDX element mapping of (**B**) carbon, (**C**) Ni and (**D**) Fe. Obtained from [118].

In alignment with Guo, Theofanidis and co-workers found the presence of amorphous and graphitic-like carbon using Raman (Figure 19) and TEM (Figure 20). Figure 19 shows the Raman spectra for graphite, a spent Fe–Ni catalyst (with Ni/(Ni + Fe) ratio of 0.6) after 1 h TOS during DRM at 1023 K, the same catalyst after CO_2_-TPO to 950 K and after CO_2_-TPO to 1123 K. The analysis for the spent Ni–Fe catalyst (black line in Figure 18) confirmed the existence of two types of carbon species structures. The G band of single crystal graphene, shifted from 1581 cm^−1^ to 1584 cm^−1^, implies the presence of graphitic-like carbon species on the catalyst (more graphene layers). According to literature, the G Raman peak changes in position, shape and intensity as a function of the number of graphene layers [130]. The D and D’ bands at 1350 and 1619 cm^−1^ were also observed and attributed to a defective and disordered structure [128,130]. This disordered carbon species structure, following from the D band, can be amorphous. The Raman spectrum of the Ni–Fe catalyst after CO_2_-TPO at 950 K (grey line in Figure 19) showed the same peaks as the spent Ni–Fe catalyst, implying the existence of the same types of carbon. Finally, the same types of carbon were observed on the Ni-Fe catalyst after CO_2_ treatment at 1123 K [118]. 

Figure 20A shows a TEM image of a spent Fe–Ni catalyst with Ni/(Ni + Fe) ratio of 0.6. The presence of filamentous carbon with Fe–Ni nano-alloy particles on top is observed, which can be verified by the EDX mapping (Figure 20B–D). 

**Figure 21:**
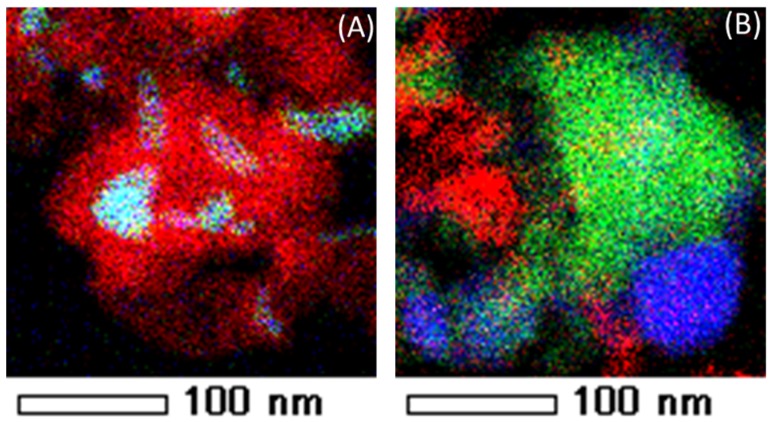
EDX element mapping of Fe–Ni. (**A**) After DRM (1023 K, CH_4_/CO_2_/He = 1.1/1/1, total pressure of 101.3 kPa, reaction time 1 h). (**B**) After CO_2_ oxidation (1 mL/s of CO_2_ at a total pressure of 101.3 kPa and 1123 K). Red, green and blue colors correspond to carbon, Fe and Ni elements respectively. Obtained from [118].

CO_2_-regeneration resulted in the removal of carbon on the active metals of the catalysts [118]. However, EDX-STEM (Energy-dispersive X-ray spectroscopy Scanning Transmission Electron Microscope) mapping (Figure 21) showed the persistence of carbon species located far from the catalyst active metals, implying the absence of direct interaction between carbon species and CO_2_ from the gas phase. 

**Figure 22:**
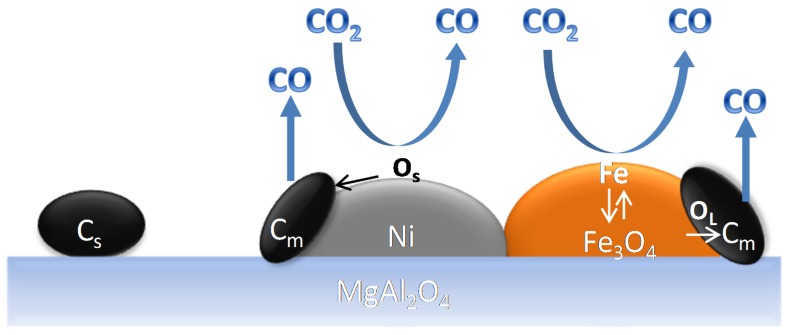
Schematic representation of carbon species removal by CO_2_ over Fe–Ni catalyst. C_s_: deposited carbon. O_s_: surface oxygen, O_L_: lattice oxygen. C_m_: carbon deposited on metals, C_s_: Carbon deposited far from metals, O_s_: surface oxygen, O_L_: lattice oxygen. The carbon illustration is not corresponding to the real carbon structure. Obtained from [118].

Theofanidis and co-workers [118] used operando XRD and isothermal experiments in a Temporal Analysis of Products (TAP) reactor, in order to unravel the major mechanistic aspects of carbon species removal by CO_2_ over a spent Fe–Ni catalyst. They reported that the process could be described by two parallel contributions (Figure 22): (1) Dissociation of CO_2_ over Ni followed by the oxidation of carbon species by surface oxygen; (2) Fe oxidation by CO_2_ and subsequent carbon species oxidation by Fe oxide lattice oxygen (Fe oxide reduction step).

### 4.2. Trimetallic Fe-Containing Alloys for Hydrocarbon Conversion

**Figure 23:**
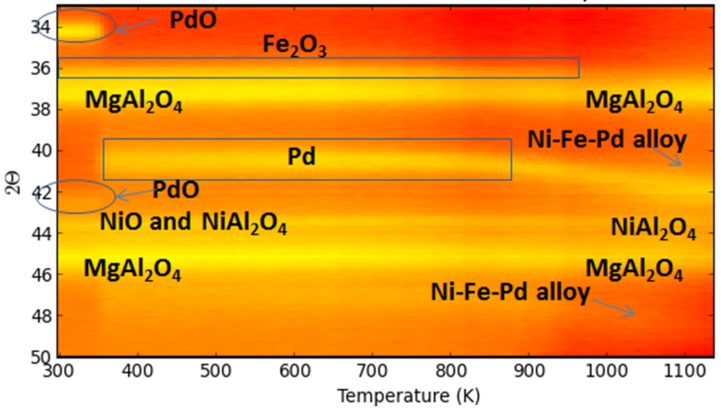
2D in situ XRD pattern during H_2_-TPR for Fe–Ni–Pd. Heating rate: 30 K/min, maximum temperature 1123 K, flow rate: 1 NmL/s, 10%H_2_/He. Obtained from [116].

A trimetallic Fe-containing alloy, along with Ni and Pd supported on MgAl_2_O_4_, forming upon H_2_-Temperature programmed reduction (TPR), was also reported by Theofanidis and co-workers [116]. Time-resolved in situ XRD (Figure 23) was used to follow up on the phases. The diffraction peaks associated to Fe_2_O_3_ were not detected due to the low concentration and their overlapping with MgAl_2_O_4_ peaks. However, during reduction, PdO peaks disappeared at 400 K and NiO peaks above 800 K. The metallic Pd related diffraction shifted from 40.1° to an angle of 42.4°, above 820 K, higher than that for Ni–Pd alloy (41.9°), which was hence attributed to a trimetallic Fe–Ni–Pd alloy diffraction peak [116]. 

**Figure 24:**
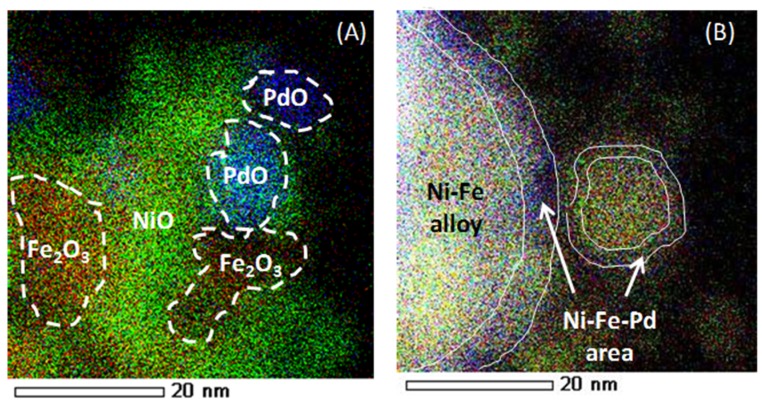
EDX element mapping of a Fe–Ni–Pd catalyst supported on MgAl_2_O_4_. (**A**) as-prepared (**B**) reduced (1 NmL/s of 5%H_2_/He mixture at a total pressure of 101.3 kPa and 1123 K). Red, green and blue colors correspond to Fe, Ni and Pd elements, respectively. Obtained from [116].

The elemental distribution of “as-prepared” and reduced Fe–Ni–Pd catalyst is indicated in Figure 24, using energy-dispersive X-ray spectroscopy (EDX)-STEM mapping. Oxide clusters are detected in the as-prepared sample, Ni (green), Fe (red) and Pd (blue), while upon reduction the elements get redistributed, resulting in the formation of a trimetallic alloy in the outer shell. Based upon element loadings, the core of the alloy will be close to bimetallic Fe–Ni, while the surface contains truly trimetallic Fe–Ni–Pd [116]. The trimetallic Fe–Ni–Pd alloy with low Pd concentration, less than 0.5 wt %, has been utilized for syngas production [116], displaying promising results in terms of suppressing carbon formation due to Fe presence. The stability of Fe–Ni catalyst increases due to Pd addition by means of a thin Fe–Ni–Pd shell surface layer in the alloy. The latter acts as a barrier for Fe segregation from the core during syngas production [116].

Noble metals like Pt and Pd are good dehydrogenation catalysts that have been widely used [131,132,133,134,135,136]. The property of the aforementioned Fe–Ni–Pd catalyst to form a core-shell alloy structure after reduction, where small concentrations of Pd are mainly located in the shell, in combination with the carbon-resistance of the catalyst due to the presence of Fe, can be exploited during propane dehydrogenation (PDH) and oxidative propane dehydrogenation (OPDH). The dehydrogenation of light alkanes (ethane, propane, butane) obtained from natural gas sources is considered an important route for the selective production of high-purity alkenes, which are basic chemicals for the industry. An important industrial propylene production is based on selective, non-oxidative propane dehydrogenation resulting in catalyst deactivation, low conversion. Oxidative dehydrogenation (ODH) provides a promising alternative route based on elimination of thermodynamic limitations and avoiding of catalyst regeneration. Indeed, co-feeding an oxidant such as CO_2_ can offer a myriad of opportunities, especially for catalysts containing Fe, which has proven to suppress carbon deposition. Furthermore, the oxidant CO_2_ will react with product H_2_, thereby shifting the equilibrium and enhancing the catalyst selectivity. The by-products of the CO_2_-ODH reaction are CO and H_2_O, via the reverse water gas shift reaction. Catalysts with redox properties, such as Fe-based catalysts, could possess high catalytic activity for the various ODH reactions of hydrocarbons. 

Our preliminary results show that the addition of Pd to Fe–Ni slightly increase the selectivity of the catalyst towards the main product of C_3_H_6_, while the C_3_H_8_ conversion during propane dehydrogenation at 873 K was slightly higher compared to bimetallic Fe–Ni. On the other hand, during oxidative propane dehydrogenation, the trimetallic Fe–Ni–Pd showed slightly higher C_3_H_8_ conversion, but lower selectivity compared to Fe–Ni. 

Further optimization of the catalysts is needed in order to fine-tune the catalytic properties through alloying. Nano-alloys synthesized by mixing elements, can produce intermetallic compounds with significantly modified properties compared to the monometallic counterparts, due to “synergistic effects”. Their chemical reactivity can be changed by modifying the composition and atomic ordering, as well as the size of the clusters. This ability to modify and fine-tune properties through alloying is the reason why the field of nano-alloys in catalysis is increasingly attracting scientific attention. 

## 5. Summary and Outlook: The Role of Fe

Significant progress has been achieved in the past few years on understanding the role of Fe in nano-materials, in view of further utilization in chemical processes as a promoter or catalyst. In this review, the role of Fe, the current challenges and the future opportunities of using Fe in catalytic systems have been presented and discussed.

(1) The addition of Fe, either in bimetallic catalysts or incorporated into the support lattice, can provide redox functionality to the catalyst, which helps to suppress carbon formation.

The bimetallic Fe–Ni catalyst showed higher activity and stability compared to the monometallic samples, as the FeO_x_ species which form under reaction conditions in the presence of an oxidizing agent (CO_2_, H_2_O or O_2_), react via a redox mechanism with the carbon deposits. On the other hand, the Fe concentration is a crucial parameter for the catalytic stability, because of Fe segregation from the Fe–Ni alloy under reaction conditions. Therefore, Ni-rich catalysts with Ni/(Ni + Fe) ratio equal to or higher than 0.8 are preferred. The dosed amount of Fe can still increase the carbon-resistance of the catalyst, while, at the same time avoiding deactivation due to blocking of Ni sites.

(2) The mechanism of carbon species removal by CO_2_ over bimetallic Fe–Ni is different from that over a monometallic Ni catalyst.

Carbon deposits close to active metals can be removed by CO_2_, a process that can be described by two parallel contributions. One contains the dissociation of CO_2_ over Ni and subsequent oxidation of carbon species by the surface oxygen. The second consists of the Fe oxidation by CO_2_ followed by carbon species oxidation by Fe oxide lattice oxygen, i.e., Fe oxide reduction.

(3) The redox properties of Fe can be exploited in different processes.

The use of Fe is not limited to the processes described in this review. The super-dry reforming process was developed based on Fe redox properties. Fe_2_O_3_ supported on MgAl_2_O_4_ was used as a solid oxygen carrier material and three molecules of CO_2_ were consumed per one CH_4_, resulting in an enhanced CO production. Because of the multiple oxidation states of Fe, Fe–Ni alloys were also exploited as oxygen carriers during chemical looping dry reforming, tuning the product selectivities when CH_4_ is used as a fuel.

The novel MgFe_x_Al_2−x_O_4_ support, where Fe is incorporated in the octahedral sites of the magnesium aluminate spinel structure can be further optimized and exploited as a new, low cost support material for different processes. The redox functionality acquired by the Fe addition to magnesium aluminate combined with enhanced thermal stability are required properties that a support material should offer. Further insight in catalyst optimization, in terms of activity and stability, can be obtained by investigating the oxygen mobility of this material when a metal, such as Ni, is deposited on top of the MgFe_x_Al_2−x_O_4_ support.

## Figures and Tables

**Table 1:** Segregation energies without (Δ*E_seg_*, kJ/mol) and with adsorbates ($∆E_{seg}^{ads}$) for the exchange of Fe in the subsurface layer of a (111) surface of Ni_3_Fe or Ni_2_FePd, with Ni or Pd from the surface layer, for various coverages, representative for methane dry reforming (DRM). All coverages refer to the species adsorbed on the fcc sites of a periodically repeated unit cell with 4 surface atoms. Obtained from [116].

Δ*E_seg_* (kJ/mol)	Ni_3_Fe	Ni_2_PdFe	
Adsorbate Overlayer	Fe ↔ Ni	Fe ↔ Ni	Fe ↔ Pd
0% (vacuum)	+55	+53	+104
100% H	+49	+52	+43
100% CO	+29	+42	+3
100% O	−94	−76	−218
50% CO, 50% O	−25	−8	−92
50% CO, 25% O, 25% H	+1	+11	+38
25% of CH, CO, O, H	+2	+9	+143
